# γ-H2AX Foci Persistence at Chromosome Break Suggests Slow and Faithful Repair Phases Restoring Chromosome Integrity

**DOI:** 10.3390/cancers11091397

**Published:** 2019-09-19

**Authors:** Michelle Ricoul, Tamizh Selvan Gnana Sekaran, Patricia Brochard, Cecile Herate, Laure Sabatier

**Affiliations:** PROCyTox, French Alternative Energies and Atomic Energy Commission (CEA), Paris-Saclay University, 92260 Fontenay-aux-Roses, France; michellericoul@free.fr (M.R.); tamizh096@gmail.com (T.S.G.S.); patricia.brochard@cea.fr (P.B.); cecile.herate@cea.fr (C.H.)

**Keywords:** chromosome, premature chromosome condensation, γ-H2AX, dicentric chromosome, DNA repair, telomere, irradiation

## Abstract

Many toxic agents can cause DNA double strand breaks (DSBs), which are in most cases quickly repaired by the cellular machinery. Using ionising radiation, we explored the kinetics of DNA lesion signaling and structural chromosome aberration formation at the intra- and inter-chromosomal level. Using a novel approach, the classic Premature Chromosome Condensation (PCC) was combined with γ-H2AX immunofluorescence staining in order to unravel the kinetics of DNA damage signalisation and chromosome repair. We identified an early mechanism of DNA DSB joining that occurs within the first three hours post-irradiation, when dicentric chromosomes and chromosome exchanges are formed. The slower and significant decrease of ”deleted chromosomes” and 1 acentric telomere fragments observed until 24 h post-irradiation, leads to the conclusion that a second and error-free repair mechanism occurs. In parallel, we revealed remaining signalling of γ-H2AX foci at the site of chromosome fusion long after the chromosome rearrangement formation. Moreover there is important signalling of foci on the site of telomere and sub-telomere sequences suggesting either a different function of γ-H2AX signalling in these regions or an extreme sensibility of the telomere sequences to DNA damage that remains unrepaired 24 h post-irradiation. In conclusion, chromosome repair happens in two steps, including a last and hardly detectable one because of restoration of the chromosome integrity.

## 1. Introduction

Scoring of chromosome rearrangements is largely applied to determine and quantify genotoxic effects of environmental components, pharmaceutical drugs, or ionizing radiation. Indeed, clastogenic effects of such agents will lead to double strand breaks (DSBs) and later chromosome rearrangements during the DNA repair phase. The cells are employing different mechanisms in order to repair the DSB depending on the cell cycle phase, and this can lead in some cases to chromosome aberrations (CA). We focus on the exchange-type aberrations, i.e., dicentrics and translocations. The dicentric chromosomes result from the fusion of two broken chromosomes with one centromere, and thus finally contain two centromeres. It is usually associated with acentric fragments without centromere. The translocated chromosomes result from the association of two chromosomes restoring an apparent structure integrity (one centromere and telomeres at each extremity). To evaluate DNA DSB formation, a main surrogate is the following and scoring of γ-H2AX foci. However, the kinetics of the γ-H2AX foci appearance and disappearance as well as the CA formation remain poorly understood.

Both classes of the detectable CA are caused by DNA DSBs, critical damage of DNA leading to genetic instability, genome mutation, and negatively affecting individual survival. Rapidly after DSB, the DNA damage response (DDR) is initiated. An early biomarker of DSB is the phosphorylation of the C-terminal part of the histone H2AX that occurs on the Serine 139 within a Serine/Glutamine motif during the seconds following DNA damage [1]. This rapid and transient phosphorylation of H2AX can be catalysed by different phosphoinositide 3-kinase-related protein kinases (PIKK) such as Ataxia Telangiectasia Mutated/Ataxia Telangiectasia and Rad3-related proteins (ATM/ATR) [2,3]. In the case of ionising radiation, ATM is preferentially recruited to induce H2AX phosphorylation, thus activating the recruitment and the retention of a cascade of DDR proteins, through the mediator of the DNA damage checkpoint (MDC1) involved in the recruitment of the p53binding protein 1 (53BP1) [4,5]. Both MDC1 and 53BP1 trigger the binding of the MRE11-RAD50-NBS1 (MRN) complex [6]. Phosphorylation of H2AX is a key step in the process of DSB repair and is a well characterized biomarker for dose exposition assessment with characteristic and well-studied two-phase kinetics [7,8]. As a proof of concept, H2AX−/− mice were to be radiation-sensitive and displayed an important level of abnormal CA [9].

Indeed, the γ-H2AX kinetics has been widely explored in different cell types both in vivo and in vitro [10,11,12,13,14]. The appearance of γ-H2AX foci occurs quickly after radiation exposure and is detectable within the first minutes post-exposure. The level of the γ-H2AX foci reaches a maximum during the first 10 min to 30 min post-exposure and declines to background level within 24 h [1,15,16,17,18]. This signalling has already been quantified, with 1 Gy of irradiation leading to 1% of H2AX phosphorylation and 35 DSBs. Thus, the number of foci will be 35, since the γ-H2AX foci number is considered to reflect exactly the DSB rate [1]. Nevertheless, the kinetics seems to be dependent on the tissue type, especially in non-dividing mammalian cells [14]. The mechanism of γ-H2AX dephosphorylation also remains quite unknown, since two hypotheses coexist stating that either the γ-H2AX is directly dephosphorylated by phosphatase or that γ-H2AX histones are replaced by non-phosphorylated molecules [17]. The rapid return to the background levels about 24 h post-exposure is also quite controversial, since some studies report the detection of γ-H2AX after DNA damage from days to months [19]. However a widely accepted consensus is that 80% of DNA DSBs are repaired during a fast phase occurring the first few hours post-exposure, while the remaining 20% are repaired later during a second slower phase [18,20]. For example, in lymphocytes, 7 h after 4 Gy exposure only 25% of γ-H2AX foci are still remaining [21,22]. That is why γ-H2AX foci are frequently scored as a surrogate for DNA DSBs.

DNA DSBs are repaired by four distinct pathways depending on the cell cycle phase: non-homologous DNA end joining (NHEJ), alternate end joining (a-EJ), homologous recombination (HR), and single-strand annealing (SSA). While HR operates rather an accurate repair, the three other mechanisms are mutagenic and induce DNA sequences loss [23]. The appearance of DNA lesions (about 30 base pairs (bp)) and chromosomal rearrangements (more than 1000 bp), such as dicentric chromosomes, translocations, or large deletions has been extensively debated. Chromosomal rearrangements are formed when two DSBs are mis-joined. Based on the idea of a “contact first”, the joining of two broken chromosomes takes place when the breaks are located in a proximal position. If the clustered DSBs occur at the chromosome boundary, this will probably lead to interchromosomal exchange [24,25].

Here, we have developed a new method to study in parallel the chromosome localisation and the kinetics of the γ-H2AX appearance and disappearance, as well as CA formation after a genotoxic event. To induce DNA DSB, cells were irradiated with γ-rays of 2 or 4Gy that will break the DNA by a well-controlled hit-and-run mechanism. For the analysis of chromosomes repair, we associated the classic Premature PCC method [26,27] with a cytospin chromosome spreading and a fixation method that preserve the chromosome-associated proteins. This protocol will allow, on each cell, first for immunofluorescence of γ-H2AX and then for the detection of the different CA after telomere and centromere FISH (Fluorescence In Situ Hybridization) staining [28] or Multi-FISH (M-FISH) staining. γ-H2AX foci, considered as a highly reliable DNA DSB marker, remain 8 and 24 h post irradiation on the site of the DSB and can be present even after chromosome fusion and repair. More precisely, we determined that slow and error-free rearrangement can occur until 24 h post-DNA break while dicentric chromosomes and translocation formation have already occurred 3 h after DNA DSB. Moreover, we identified an important quantity of γ-H2AX foci in telomere sequences before and after irradiation (IR), suggesting sequence fragility or mis-repairing of the chromosome extremities. In regards to biodosimetry, we confirmed the PCC method can be used in order to assess the received dose directly after accidental radiation exposure and further analysis after a telomere and centromere FISH staining allows for dicentric chromosome scoring.

## 2. Results

Ionising radiation exposure leads to immediate and clustered DNA lesions, chromosome single-strand break (SSB), and DSB, that are more or less quickly repaired by the cellular machinery [29]. Due to the timing of reparation, the study of the DSB repair mechanism kinetics needs an immediate visualisation of condensed chromosomes after break induction while irradiated peripheral blood lymphocytes (PBL) are mainly in the G0 phase [26]. The PCC assay allows for an immediate processing of damaged cells without requiring time for lymphocytes simulation and cell culture. The mandatory minimum delay of about 3 h is due to the fusion induction of PBL with mitotic cells for chromosome condensation, as well as for the KCL treatment and cytospin spreading.

**Figure 1:**
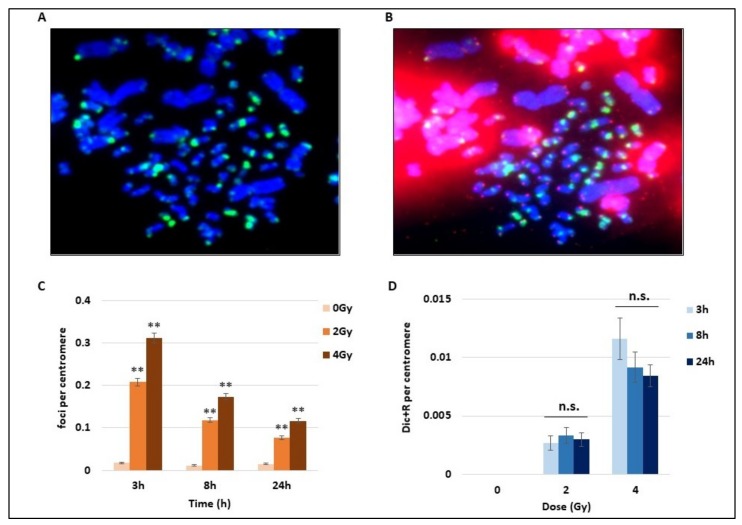
Kinetics of DNA breaks signalling and unstable chromosome aberrations after radiation exposure. Blood samples from three donors were irradiated at 2 or 4 Gy or kept free from ionising radiation, analysed using the PCC technique and fixed 3 h, 8 h, or 24 h later. (**A**) Cells were first stained by immunofluorescence with a FITC (fluorescein isothiocyanate)-coupled γ-H2AX antibody (in green) used to detect foci on both human (1-chromatid chromosomes) and hamster chromosomes (2-chromatid chromosomes). (**B**) A second staining using PNA-FISH (Peptide Nucleic Acid Fluorescence In Situ Hybridisation) Telomere and Centromere probes was performed on the same Premature Chromosome Condensation (PCC) cells after the immunofluorescence to simplify the detection of chromosome unstable aberrations (Dicentric and Ring chromosomes and Acentric fragments). Centromeres are stained in green, while telomeres are in red, DNA appears in blue after Dapi staining. Dicentric chrosomes are chromosomes with two centromeres in green. (**C**) The number of γ-H2AX foci on human chromosomes was counted after immunofluorescence staining (cf image A) and expressed per centromere to exclude bias due to the possible loss of PCC fragments during the cytospin step. The increase of foci with 2 and 4 Gy irradiation (IR) is significant at 8 h and 24 h post-IR (** *p* < 0.01) and the bars represent standard error. The number of foci decreases with time. (**D**) Dicentric and ring chromosomes were counted after Telomere and Centromere FISH staining (as represented in **B**) and expressed per centromeres. The unstable chromosome aberrations increase with doses. There is no statistical difference between the different time points at 2 and 4 Gy (n.s).

Using the whole blood of three donors, we studied the DNA damage consequences of 2 and 4 Gy of γ-radiation exposure. After the mitotic CHO cells induced PCC assay and following the cytospin spreading of cells, we performed for the first time an immunofluorescence assay on PCC. The direct γ-H2AX detection by immunofluorescence with a FITC-coupled antibody targeting γ-H2AX foci on PCC after IR is shown in Figure 1A. Abbreviations used in this figure and others are explained in the section “Abbreviations”. γ-H2AX foci are considered as early DNA DSB signalling both on hamster and human PCC (Figure 1A). On the same PCC cells, the FISH staining of telomere and centromere was performed to visualize centromeres in green and telomeres in red and easily detect unstable chromosome aberrations (Figure 1B). An unbroken human chromosome would contain only one chromatid with one centromere in the middle and one telomere at each extremity, while hamster chromosomes are mitotic and present only telomere staining. To compare the time course of γ-H2AX signalling as well as the Dic+R frequency, the numbers of foci and Dic+R were calculated per centromere. Indeed the cytospin spreading could lead to bursting of some PCC and the loss of some PCC fragments, while the telomere staining presents the disadvantage of binding to hamster intra-chromosomal sequences and making the analysis more difficult. After 2 or 4 Gy radiation exposure, an important and significant increase of γ-H2AX foci, dependent on the dose, was detected as soon as 3 h post-exposure (Figure 1C). Nevertheless, this signalling decreases quickly after 3 h post-exposure but a consequent part of residual foci was still observed 24 h post-exposure (about 25% of the total foci that were observed after 3 h). On the same cells, unstable aberrations (Dic+R frequency) were scored and as expected, the Dic+R frequency increases drastically with dose (an increase of about five-fold between 2 and 4 Gy at 3 h) but remains stable with time from 3 h to 24 h post-exposure (mean for the three times was 0.003/centromere for 2Gy and 0.01/centromere for 4 Gy). Dicentric chromosome formation seems to be an early event after DNA damage and does not progress after 3 h, while γ-H2AX foci display a different time course with an early formation and a strong decrease within the first 24 h.

**Figure 2:**
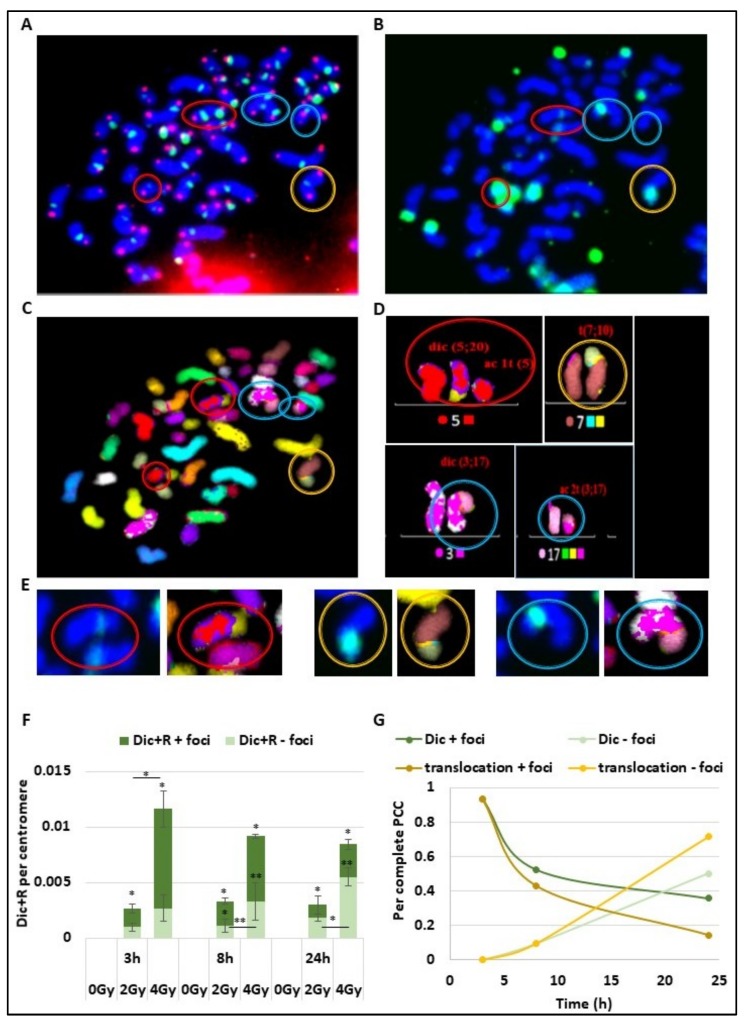
γ-H2AX foci signalling of unstable and stable aberrations short time after irradiation. (**A**–**D**) The same slide with PCC lymphocyte cells previously irradiated with 4 Gy was observed after telomere (in red) and centromere staining (in green) (**A**), γ-H2AX immunofluorescence assays have been performed with a FITC-coupled antibody (green) (**B**) or multi-FISH (**C**) and analysed with the ISIS software. Four abnormalities were detected and marked with coloured circles. The red and blue circles show dicentric chromosomes and their corresponding acentric fragments. The yellow circle shows a translocation between chromosomes 7 and 10. (**D**) Chromosome karyotype using false colours was built using the ISIS software. Data obtained from Telomere and Centromere staining and from multi-FISH were assembled to detect unstable and stable aberrations. (**E**) Zoom of CA stained for γ-H2AX (green) and visualized after multi-FISH. The foci localized at the junction between two chromosomes. (**F**) Blood samples from three donors were irradiated at 2 or 4 Gy or kept free from ionising radiation before analysis using the PCC technique. Dic+R frequency per centromere was scored 3 h, 6 h, and 24 h after IR and the percentage of co-staining with γ-H2AX foci was determined (dark green). The increase of “Dic+R + foci” or “Dic+R – foci” is significant (* *p* < 0.05, ** *p* < 0.001) at the different time points compared to 0 Gy. There is significant differences when indicated between 2 Gy and 4 Gy irradiation (* *p* < 0.05, ** *p* < 0.001). (**G**) Only complete PCC cells (between 45 and 48 chromosome fragments) irradiated with 4Gy were analysed by multi-FISH after γ-H2AX foci immunofluorescence assay. Proportions of Dic+R and translocations signalled with γ-H2AX foci (in green part A) or without any γ-H2AX foci were represented over time. Respectively 15, 21, and 14 PCC were analysed for 0, 2, and 4 Gy exposure.

To understand the time course of DSB signalling and chromosomal aberration formation due to error-prone repair, the same cells were observed after telomere and centromere staining for Dic+R detection (Figure 2A), γ-H2AX staining (Figure 2B), and multi-FISH staining (Figure 2C), using all the data the karyotype of the cells were then established (Figure 2D). On the PCC obtained after 4 Gy irradiation (Figure 2A–D), different CA were detected and marked with coloured circles. The red circles show a dicentric chromosome and the associated acentric fragment, both signalized by γ-H2AX foci. The yellow circle corresponds to a translocation between chromosome 7 and 10, also signalized with γ-H2AX foci.

The last blue circles reveal a dicentric chromosome and its acentric fragment with only a γ-H2AX signalling on the dicentric chromosome. We observe for the observed CA a persistent γ-H2AX signalling at the chromosome junction even after chromosome joining and repair (Figure 2E). Both for dicentric chromosomes and translocated chromosomes, the yellow star corresponding to γ-H2AX foci localized at the junction even after chromosome ends joined. As previously described, the Dic+R frequency increases in a dose dependent manner (Figure 2F). At 3 h post-exposure, even if they have already fused, the majority of dicentric chromosomes are still marked with γ-H2AX foci, and only 37% and 23% of Dic+R are not signalled respectively after 2 and 4 Gy of radiation exposure. However, the proportion of non-signalled unstable aberrations increases over time to reach more than 60% of Dic+R at 24 h post-exposure (respectively 61% and 65% for 2 and 4 Gy). However, about 30% of Dic+R contain persistent γ-H2AX foci several hours post-fusion (24 h) and even though the repair is ended. Focusing on complete PCC only (between 45 and 48 fragments) from PBL exposed at 4 Gy (Figure 2G), it was established that the signalling of translocations by γ-H2AX follows the same dynamic as Dic+R. Twenty-four hours after IR, 20% of translocations are still signalled by γ-H2AX foci at the junction between the 2 chromosomes.

**Figure 3:**
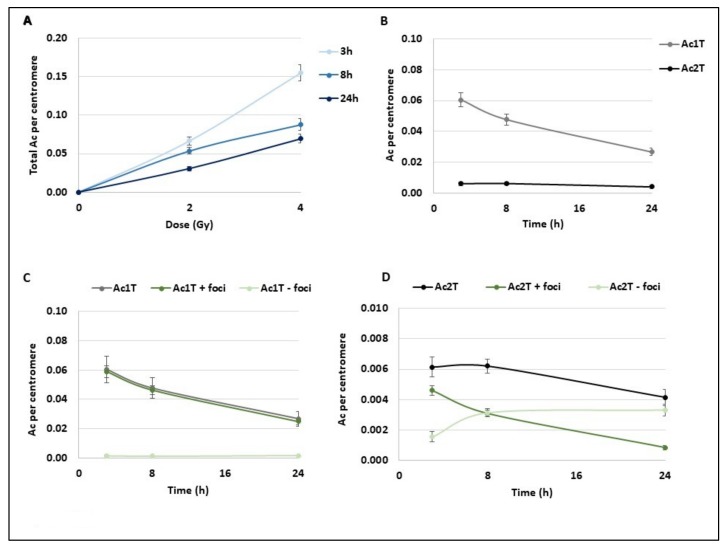
Kinetics of different subtypes of acentric fragments after radiation exposure. Blood samples from three donors were irradiated at 2 or 4 Gy or kept free from ionising radiation and analysed using the PCC technique. (**A**) After Telomere/Centromere staining on PCC, the total number of acentric fragments per centromere was scored for each dose and 3 h, 8 h, and 24 h post-IR. The total acentric fragments increase with dose and decrease overtime. Bars represent standard deviation. (**B**–**D**) Focus was made on PCC analysis after 2 Gy radiation exposure. Bars represent standard deviation. (**B**) Acentric fragments containing 1 telomere or resulting from fusion and containing 2 telomeres were separately represented at 3 h, 8 h, and 24 h post IR. A decrease of both types of acentric fragments is observed overtime (**C**,**D**) All acentric fragments were counted and classified in two classes, acentric fragments signalled by γ-H2AX foci or without γ-H2AX signal. Their proportions were followed at 3 h, 8 h, and 24 h post IR. Acentric fragments with 1 telomere resulting from one DNA DSB (**C**) and acentric fragments with 2 telomeres resulting from two DNA double strand breaks (DSBs) and fusions of two fragments (**D**) were represented separately.

Chromosome breaks lead both to unstable (Dic+R) and stable aberrations (translocations) and also to the generation of acentric fragments that are the remaining chromosome fragments without centromere. These fragments initially appear as a 1 telomere fragment (Ac1T) and usually fuse to another one to form a 2 telomeres fragment (Ac2T) that is usually not transmitted to the daughter cells. First, we followed the acentric frequency (number of acentric fragments per centromere) in the same condition as previously described (Figure 3A). Like Dic+R, the number of acentric fragments increases with dose and they are directly provoked by IR. However, they evolve differently with time and decrease significantly within the first 24 h post-exposure (Figure 3A). Surprisingly, after 2 Gy exposure, both Ac1Ts and Ac2Ts decrease with time until 24 h (Figure 3B) suggesting that Ac1Ts do not fuse together to form Ac2T. Ac1T results from one chromosome break and contains a free extremity without a telomere that is almost systematically signalled by a γ-H2AX foci at the different time points (Figure 3C) when Ac2T displays the same signalling kinetics as Dic+R and translocation (Figure 3D). At 3 h post-exposition, almost 75% of Ac2Ts are detected by γ-H2AX foci and this signalling decreases to 25% at 24 h. While both Ac1T and Ac2T frequency decreases during the 24 h post 2 Gy exposition, the signalling of both types of acentrics differs. Ac1T could still be considered “in repair” process with a free-telomere end that could potentially still fuse to restore the chromosome integrity.

**Figure 4:**
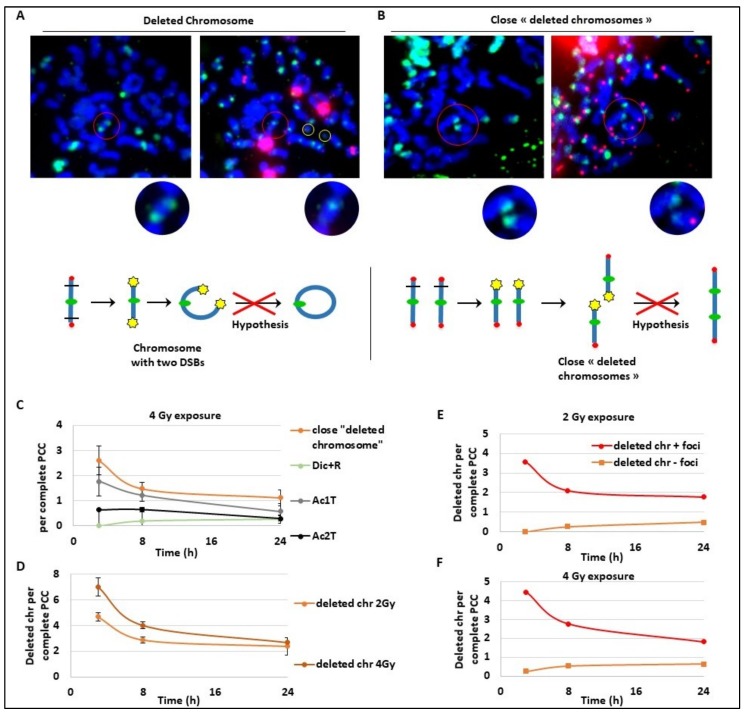
Kinetics of late and slow DNA repair mechanisms. Blood samples from three donors were irradiated at 2 or 4 Gy or kept free from ionising radiation, analysed using the PCC technique and fixed 3 h, 8 h, or 24 h post-exposition. (**A**,**B**) Images of the same PCC exposed to 4Gy were observed after γ-H2AX immunofluorescence staining (in green on the left panels) and after TC staining (right panels). Centromeres are stained in green and telomeres in red after PNA-FISH staining. Red circles show the zones of interest and yellow circles indicate acentric fragments. A chromosome with two DSBs (**A**) and two chromosomes with close signalled DSBs (**B**) are both signalled by γ-H2AX and considered as “in repair”. In (**A**), the chromosome is expected to become a ring while in (**B**), the two chromosomes are expected to fuse and make a dicentric chromosome. Below each image, a schema represents the hypothetical mechanism of CA formation. On the schema, centromeres are represented with green circles, telomeres with red circles, and γ-H2AX foci with yellow stars. The red crosses on the hypothesis conclude that the data will not confirm the hypothesis. (**C**–**F**) Scoring was performed only on complete PCC cells containing between 45 and 48 fragments. (**C**) The frequency of “close deleted chromosomes”, Dic+R, Ac1T, and Ac2T, per PCC were reported at 3 h, 8 h, and 24 h after 4 Gy irradiation. (**D**) The frequency of deleted chromosomes (with one telomere only) was followed after 2 and 4 Gy irradiation at three time points: 3 h, 8 h, and 24 h post-IR. (**E**,**F**). The proportion of deleted chromosomes containing γ-H2AX signalling or not signalled was quantified after 2 Gy (**E**) and 4 Gy (**F**) exposure.

These last results may suggest an unexpected kinetics for Ac1T and we explored deeper the future of these fragments that seem to slowly disappear, suggesting a fusion during a later phase. As a strategy, we followed both Ac1T and all unrepaired broken chromosomes containing a free end without telomeres signalled by γ-H2AX foci. On different PCC (Figure 4A,B), we detected two extremity-broken chromosomes (Figure 4A) or close deleted chromosomes due to one break (Figure 4B). The free ends of each chromosome are strongly marked by γ-H2AX foci that could suggest a late ongoing repair process. In Figure 4A, we can hypothesize the future formation of a ring with one centromere and without telomeres, and in Figure 4B we can hypothesize the fusion of the two ends of the close deleted chromosomes. We also identified Ac1T and Ac2T coming from these deletions among the PCC fragments that are also signalled by γ-H2AX foci (Figure 4A,B right panels). The drawing (Figure 4A,B) illustrates the two hypothetic mechanism of chromosome aberrations formation. To characterize this late repair after 4 Gy radiation exposure, we followed the “close deleted chromosomes” and Dic+R as well as Ac1T and Ac2T only on complete PCC to make sure to have all the fragments (Figure 4C). As expected during a later repair process, the frequency of “close deleted chromosomes” as well as Ac1T decreases between 3 h and 24 h. As observed before, during the same time-lapse Dic+R and translocation frequency remain stable, suggesting the possible fusion of the deleted chromosomes with their own Ac1T during a late, slow and accurate repair process. Indeed we confirm that after 2 Gy or 4 Gy dose exposure, the “deleted chromosome” number decreases until 24 h post-exposure (Figure 4D). We have previously observed on complete PCC the stability of dicentric chromosomes that are formed early during the repair process, and here we observe the slight decrease of Ac2T (Figure 4C). It was also pointed out that γ-H2AX signalling of “deleted chromosomes” decreases during 24 h post-exposure while undetected “deleted chromosomes” remain stable and seem to not be repaired (Figure 4E,F). In regards to this data, the hypothesis of Figure 4A and 4B are rejected. These data could rather suggest two parallel kinetics of DSB DNA repair. Dicentric chromosomes and translocations are formed early in the 3 h following DNA damage but the new chromosome structures are “incorrect”, while fusion of “deleted chromosomes” with its original fragments seems to be repaired correctly later until 24 h post-exposure.

**Figure 5:**
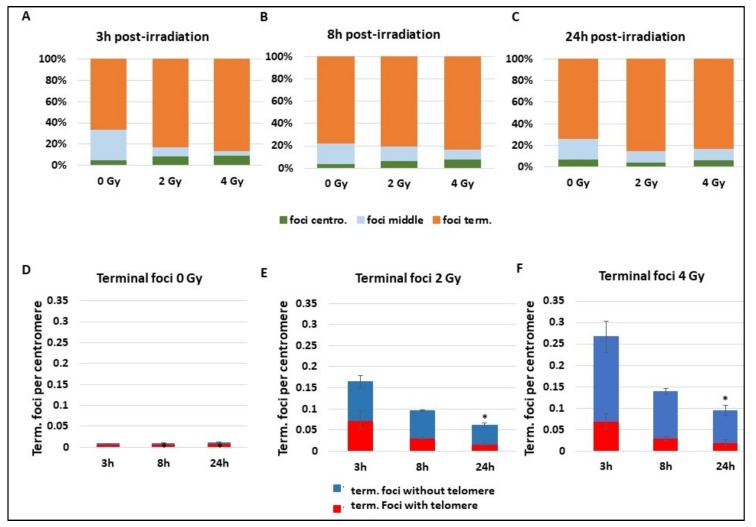
Localization of γ-H2AX foci on chromosomes. Blood samples from three donors were irradiated at 2 or 4 Gy or kept free from ionising radiation and then analysed using the PCC technique. (**A**–**C**) After immunofluorescence staining, γ-H2AX foci position on chromosome sequence was determined “close to chromosome extremities”, “close to centromere”, or “in the middle of the chromatide”. Analyses were performed at 3 h (**A**), 8 h (**B**), or 24 h (**C**) after radiation exposure. (**D**–**F**) Only γ-H2AX foci “close to chromosome extremities” were sub-classed in two groups, γ-H2AX foci associated “with telomeres” or “without telomere” and followed at 3 h, 8 h, and 24 h post radiation exposure. The graphs show the repartition of foci without irradiation (**D**) and after 2 Gy exposure (**E**) or 4 Gy (**F**) radiation exposition. The decrease of terminal foci without telomere is significant at 24 h (*p* < 0.05).

Whatever the repair mechanism occurring, DNA DSBs are detected early by γ-H2AX. A focus on γ-H2AX foci reveals a background localization (0 Gy) of foci on terminal extremity of chromosomes containing the telomeres, with about 80% of foci on the terminal zone (Figure 5A–C). Radiation exposure of 2 and 4 Gy does not change the γ-H2AX foci localization, which remains for 80% of them on the terminal part of the chromosome. Statistical tests do not reveal any differences. Focusing on γ-H2AX foci localized on terminal extremities of chromosomes reveals an important localization of more than 80% of foci at telomere sequences at background levels (Figure 5D). There is quite a disproportion between the percentage of foci on the telomere and subtelomere sequences and the length of these zones compared to the whole genome size. First, this suggests an important occurrence of DNA breaks signalling during the maintenance process of telomeres, or it can be a recruitment of γ-H2AX through another mechanism. When cells are exposed to 2 and 4 Gy, γ-H2AX foci drastically increase and an important part of the terminal foci appears to signal chromosome extremities without telomere that are in fact DNA DSBs (“deleted chromosomes”) (Figure 5D,E) (*p* < 0.05 for the frequency of terminal foci without telomere at 2 Gy 3 h and 4 Gy 3 h compared to 0 Gy 3 h). At 3 h and 8 h after 2 Gy IR, more than 30% of foci are still present on telomere or subtelomere sequences that still only represent a small part of the total genome. The extensive signalling of telomere sequences after radiation reveals a particular sensibility of these sequences that could be misrepaired or more sensitive to IR. Indeed, even if the proportion of γ-H2AX foci in telomeres decreases after IR, the absolute number of foci increases between 0 and 2 or 4 Gy (*p* < 0.01 3 h post IR), suggesting important damages in these regions.

## 3. Discussion

γ-H2AX foci are usually quantified by immunofluorescence assays on the interphasic nucleus, providing no evidence of foci localization at the chromosome scale [30]. Here, we established a novel method by adapting the conventional PCC assay for biodosimetry with G0 primary lymphocytes to perform an immunofluorescence assay on cytospin-spread and condensed interphasic chromosomes. Such a method has previously been described by the group of A. Genesca for metaphasis spreads analysis in order to study DSB signalisation in ATM-deficient cells [31] and successfully used in our lab to identify lead effects on telomeres [32]. In this study, our described protocol has been conceived and tested in the laboratory for γ-H2AX immunofluorescence detection on PCC and could easily be adapted to other nuclear protein studies, paving the way to further investigations on chromosome-associated proteins and repair mechanisms. The advantages of the technique are multiple. There is no delay due to cell culture and as a consequence, it allows for the visualization of most of the chromosome damage that could be lost during the cell culture time [33]. Moreover, and contrary to the majority of the existing studies, the γ-H2AX foci can be counted and localized on the chromosomes.

In this study, we first follow the kinetics of stable and unstable aberrations after IR, which is used here to induce a brief and well-controlled genotoxic stress event. A dicentric and rings quantification was performed using the telomere and centromere FISH staining, while complex chromosome exchanges (translocations) were followed with M-FISH. We validated the potential use of G0 PCC for intra- and inter- chromosome aberrations for immediate CA detection, as recently published [28,33,34]. More precisely, we report that total unstable aberrations (Dic+R) reach a maximum level until 3 h post-exposure and remain stable at 8 h and 24 h post-exposure. To compare with other studies, 0.003 Dic+R/centromere is equivalent to 0.14/cells after 2 Gy and 0.01 Dic+R/centromere is equivalent to 0.46/cells for 4Gy if we consider 46 centromeres per analysed PCC. The results obtained for 2 Gy IR are in accordance with other previous studies while for 4 Gy, the frequency is two or three times lower than previously reported [28,33,34]. Moreover, we saw that Dic+R formation already occurs at 3 h post-exposure. Following 1 and 2 Gy X-rays IR, Darroudi et al. reported that dicentrics are formed initially and that the frequency remains constant [35]. However, after 4 Gy of X-rays, the Dic frequency reaches an early plateau until 3 h of post-incubation time (corresponding to 6 h post-exposure in our study) and then follows a gradual increase until a second plateau at 12–18 h (corresponding to 15 h and 21 h post-exposure in our study). However, Pantelias’ laboratory established a dose–response curve for Dic+R frequency at 8 h and 24 h post-exposure and reported no significant difference [34]. When we scored the total Dic, we did not detect any evolution from 3 h to 24 h, however, when we focused on complete PCC (Figure 4), we could detect a small increase within the same time-lapse. The kinetics of translocation formation according to our data is also quick and happens in the first hours following the [36] genotoxic exposure. While it is confirmed that for 1 and 2 Gy X-ray or γ-ray exposure, translocations remain stable from an early time, other studies detect a limited increase after 3 h following high IR doses (superior to 4 Gy) [33,35]. Recently, Biehs et al. deciphered the DNA DSB repair. Their results obtained with hTert-immortalized fibroblasts irradiated with 7 Gy X-rays confirm the biphasic kinetics of DNA DSB. They describe a first phase until 6 h when most of the DSB is repaired and a limited number of translocations are formed while in a second phase until 14 h post-IR when only 20% of repair occur, the number of translocation doubles. Indeed it seems in opposition with what we found but it is important to consider that the model is a fibroblast cell line that is supposed to be in G1 when irradiated but the protocol does not exclude that a portion of cells could be irradiated in G2 and will not undergo the S phase for repair process [36]. A study focus on DNA DSB repair in G2 phase and describe mainly a rapid kinetics of repair and CA formation [37]. The kinetics we described have to be considered only for DNA DSBs on quiescent lymphocytes and it is important to keep in mind that the rapidity of repair and CA formation can vary according to the phase of the cell cycle. Finally, in the studies focusing on blood cells, a slow and long decrease of “deleted chromosomes” or “number of fragments” until 24 h post-exposure is detected, whatever the studied dose, which ranged from 1 to 6 Gy. This suggests a slow phenomenon leading to the repair of chromosome breaks and that does not lead to CA formation [33,35]. So we suggest two steps of DNA DSB repair. The first and quick one leading to CAs followed by a slow and error-free repair mechanism. This corroborates a study showing that B-CLL cells, resistant to apoptosis after irradiation, repair DNA DSBs more quickly but show higher level of CA than radiation-sensitive B-CLL cells [38].

The kinetics of DNA DSB repair are already well described even though sometimes controversial [1,10,12,30]. While many proteins such as 53BP1 and other multiple DDR proteins are involved in the signalling and repair mechanism, here we focus on γ-H2AX foci detection to follow DNA DSB generated by IR. After an important increase of γ-H2AX foci of about 17 times at 3 h post 4 Gy irradiation, we describe a fast decrease as reported earlier [1]. However, the PCC technique does not allow an immediate visualization and quantification of γ-H2AX foci due to the delay of the fusion induction, making the quantification of the maximum level of IR-induced DNA DSB impossible. Indeed, a maximum of γ-H2AX foci are detected from 3 min post-exposure and start to decrease rapidly before 30 min due to the fast DNA DSB repair, which is initiated immediately after IR [15,18]. The γ-H2AX foci number reduction, as well as the DNA DSB repair estimated by different methods, occur in two phases. An early and important component of the kinetics happens in the first 3 h, while a late and slow mechanism of γ-H2AX foci disappearance occurs within the following hours and up to some days. Performing the PCC technique, we can only detect the second phase of the kinetics and in this case, we follow only the decrease of γ-H2AX foci corresponding to the late repair. This could explain the quite persistent and stable level of chromosome aberrations detected that are generated rapidly after damage induction. Indeed chromosome rearrangements generated by the DNA repair process do not re-establish the cell integrity, while after 3 h the late reparation does not generate new chromosome rearrangements. Our data suggest a slow process starting about 3 h post IR, where proximal broken and uncapped chromosome fragment extremities will join to restore the original chromosome.

However, here we reported persistent γ-H2AX foci at the joint of rearranged chromosome fragments until 24 h post IR. Twenty-four hours after 4 Gy IR, the level of foci is still five times higher than the background level. The persistence of γ-H2AX signalling was already shown in several studies summarized by Siddiqui et al. [19]. Residual H2AX phosphorylation was detected on fibroblasts irradiated with low and high doses in vitro as well as in in vitro irradiated lymphocytes [19]. Moreover, some patients receiving doses through different exams or treatments display residual foci after 24 h [39,40,41]. It was reported that persistent γ-H2AX foci were higher in in vitro X-ray irradiated lymphocytes of children with cancer compared to healthy children, suggesting that cancer-suffering children display impaired DNA repair capacity [42]. In addition, it was demonstrated that patients suffering from excessive normal tissue toxicity display an elevated persistent level of foci, establishing persistence of γ-H2AX signalling as a biomarker of radiosensitivity [43]. Therefore, we can hypothesize that our remaining foci could also be due to unrepaired or misrepaired DNA DSB provoked by the particularly acute and high doses received.

Here, we detected two types of persistent γ-H2AX foci, foci on broken extremities corresponding to a “late and slow ongoing repair” and foci persisting after chromosome ends joining has occurred. The first class of foci are still signalling DNA DSBs on so-called “deleted chromosomes” or Ac1T and are thus expected to disappear later. Interestingly, we demonstrated that this late repair was mainly accurate by fusing the two initially corresponding DNA extremities. This opposes to a recurrent idea that an elevated rate of late DNA DSB repair is a negative outcome and reveals acute radiosensitivity [44]. We showed that the second class of foci remains until the end of the repair process, which takes time to be completed; other events could possibly occur after the apparent ends joining. Considering the relatively high dose of exposure (2 and 4 Gy), we can hypothesize that IR could also induce an aging mechanism through a process of accelerated senescence detected here by γ-H2AX foci [12,45]. In the case of NHEJ, accelerated cellular senescence seems to correlate well with the level of genomic instability [45,46]. Thus, after important chromosome rearrangements, cells will keep the memory of abnormal changes and induce protective mechanisms or limit the replication of the damaged cells.

Our imaging approach using PCC makes possible the localisation of γ-H2AX foci and, here, an important ratio of foci was detected at chromosome extremities and in telomere sequences especially in the absence of IR. At first, two hypotheses came up: Either extremities and telomere sequences are more fragile or they are not repaired. Different studies have analysed the ratio of DNA DSBs and γ-H2AX signalling in telomere sequences. Since telomere length is a biomarker of aging and telomere shortening is one of the main mechanisms of senescence, a study linked the proportion of foci in telomeres with senescence and revealed an increase with aging in cultured cells [12]. Cells undergoing senescence harbour DNA DSB signalling at telomere sequences by recruiting DNA repair proteins, who form γ-H2AX unrepaired persistent foci [47]. Finally, a live-cell imaging study reveals that the totality of the remaining foci, after several days, is localized at telomere sequences [48]. Hewitt et al. demonstrated also that γ-H2AX localization at telomeres increases with age in a mice model, while it does not correlate with telomere length. Different scenarios have been proposed to explain the excessive signalling of telomeres. Basically, telomere and subtelomere regions could represent preferential targets for DNA damage. Indeed, their repeated sequences with guanine triplets make them more sensitive to oxidative modifications and previous work demonstrated a particular sensitivity of these regions [48,49,50]. It is also possible that genotoxic stress provoked by IR induces the loss of the telomere loop conformation and leads to a DDR formation at telomere extremities that cannot be repaired [51,52]. Cesare et al. proposed a more complex mechanism with a potential state of telomere capping able to retain TRF2 and to supress NHEJ without inhibiting DDR formation at telomere ends [53]. In fact, this last hypothesis correlates with different studies reporting γ-H2AX foci at uncapped telomeres during senescence state. Thus, senescence could even induce γ-H2AX foci without any DNA damage [54].

The analysis of induced CA due to the misrepair of DNA DSB is a powerful approach for the assessment of the genotoxic effects of exposure to ionising radiation [55]. Direct assessment of CA after radiologic accident is one the main biodosimetry methods, used particularly in the absence of physical dosimetry, to estimate the absorbed radiation dose received in the exposed population [56]. Unstable chromosome aberrations such as dicentric and ring chromosomes (Dic+R) are considered as the most sensitive method for biological dose assessment when performed on metaphasis after two days of cell culture. Indeed DSB are specific and display very low background levels [48]. However, dicentric chromosomes as well as acentric fragments are partially lost during cell division due to the mis-segregation of the two centromeres, although some dicentrics (mainly those with very close centromeres) are quite well transmitted [57]. So, the technique is mainly reliable in the short-term after exposure, making it the gold standard [58]. For long-term dose assessment, translocations, restoring an apparent integrity of the chromosomal structure and transmitted to the descendant lineage, are preferentially analysed for retrospective biodosimetry [59]. Here we focus on the PCC technique that is an alternative for immediate biodosimetry after exposure and displays advantages in case of very high dose exposure [60,61]. In biodosimetry, the official PCC protocol suggests performing the analysis in the following hours after exposure, by counting the excess of chromosome fragments [61,62]. According to our results, the number of acentric fragments evolves within the first 24 h and the counting should rather be done after this delay. Previous studies had already suggested coupling PCC with chromosome painting in order to detect stable aberrations and evaluate the received dose [63,64]. In our study, we provide evidence that dicentric formation occurs quickly after exposure. Performing the PCC technique coupled with a telomere and centromere FISH staining and evaluating the dose by dicentric chromosome scoring appears to be a reliable approach for the hours following the irradiation.

## 4. Materials and Methods

### 4.1. Lymphocyte Isolation and Irradiation

Peripheral blood was obtained from three healthy blood donors after informed consent and was collected in heparin tubes. Whole blood was kept at room temperature until irradiated and further processed. The whole blood was irradiated at room temperature with ^137^Cs γ-rays of 2 and 4 Gy and at a dose rate of 0.76 Gy/min. Then, the lymphocytes used in this study were isolated applying the commonly used Ficoll isolation method. The isolated lymphocytes were resuspended in RPMI-1640 supplemented with 10% fetal calf serum (FCS).

### 4.2. Premature Chromosome Condensation

Premature chromosome condensation was performed as previously described [28] using CHO cells. Briefly, CHO cells were grown in DMEM/F12 (1:1) medium supplemented with 10% FCS, 1% L-glutamine, and antibiotics and incubated at 37 °C in a humidified atmosphere with 5% CO_2_ in monolayer cultures in plastic T75-cm^2^ flasks. Colcemid (KaryoMax Gibco, New York City, NY, USA) was added at a final concentration of 0.1 µg/mL to the cell culture to obtain mitotic cells, followed by a further 4 h incubation at 37 °C. The accumulated mitotic cells were harvested by the shake-off method. The mitotic cells were then kept in ice until the fusion step with interphase cells. Cell fusion and the induction of PCC with polyethylene glycol (PEG) were performed as previously described [28]. Mitotic CHO cells and irradiated lymphocytes were mixed in 15-mL round-bottom culture tubes in the presence of colcemid. After centrifugation, the supernatant was discarded and 150 µL of 50% (w/v) PEG prepared in PBS was gently added by inverting the tubes. Subsequently, 1.5 mL of RPMI-1640 medium without FBS and complemented with colcemid (0.1 µg/mL) was slowly added and the cell suspension was centrifuged. The supernatant was discarded, and to initiate the fusion, the cell pellet was re-suspended in 0.7 mL pre-warmed RPMI-1640 medium supplemented with 10% FCS, colcemid (0.1 µg/mL), and 2% phytohemagglutinin, and incubated for 100 min at 37 °C.

### 4.3. Cells Spreading and Fixation

At the end of fusion, 3 mL of prewarmed KCl 0.075 M solution was added and cells were incubated for 5 min at RT. The PCC spreading was done using the cytospin technique on poly-Lysine slides and ideally 200 µL of PCC cells were placed in each well (cytofunnel). After 12 min of 1200 rpm cytospin with low acceleration, the cells were fixed for 5 min in a fixative solution (PFA 4%/2% sucrose/triton 0.1%). The total procedure from the Ficoll isolation to the cell fixation requires 3 h.

### 4.4. Immunofluorescence Assay

Cells were first permeabilized for 10 min in a solution containing 20 mM Tris-HCl pH 8, 50 mM NaCl, 3 mM MgCl_2_, 0.5% Triton X100, and 300 mM sucrose and blocked overnight at 4 °C in PBS1X containing BSA 4% and milk 2%. PCC were stained with a 488 Alex-Fluor anti-γ-H2AX antibody (Merck-Millipore 05-636-AF488, Darmstadt, Germany) diluted (1/400) in antibody buffer (PBS 0.8% containing 50 mM NaCl, 0.5% Triton X-100, and 3% milk) during 1 hour at 37 °C. Then the cells were rinsed for 8 min with antibody buffer without milk and again for 5 min with PBS1X. Before mounting, DNA was counterstained for 5 min with DAPI and rinsed with PBS.

### 4.5. Telomere and Centromere FISH Staining (TC Staining)

Telomere and centromere staining of PCC lymphocytes was performed by FISH using a Cy3-labeled PNA probe specific for telomere sequences and a FAM-labeled PNA probe specifically for centromere sequences (Panagene, Daejeon, Korea). Briefly, after immunofluorescence staining and image acquisition, the stored slides were incubated 30 min in PBS to facilitate the coverslip removal and then washed for 20 min in PBS/Tween. PCC fusions were fixed for 5 min in Ethanol/Acetic acid (3/1, *v*/*v*), washed twice in PBS for 5 min and digested in a prewarmed pepsin solution (5 mg/100 mL) for 3 min at 37 °C. Finally the cells were refixed using 4% formaldehyde for 2 min. After washing three times with PBS, the slides were dehydrated with 50, 70, and 100% ethanol for 5 min and air dried for 1 h at RT. PNA probes (0.3 µg/mL) for TC staining were applied co-denatured for 3 min at 80 °C, and the slides were incubated for 2 h in a humidified chamber at RT in the dark. After hybridization, the slides were washed as previously described [28] and counterstained with DAPI (1 µg/mL) and anti fade mounting medium.

### 4.6. Multi-FISH Staining

Staining was performed using multicolour FISH probes (MFISH 24XCyte, MetaSystems Hard & Software GmbH, Altlussheim, Germany) according to the protocol provided by the manufacturers on the same slides used for γ-H2AX immunofluorescence and TC staining. Images of PCC cells were captured using the image Metasystems Metafer/AutoCapt software.

### 4.7. Slide Scanning and Image Acquisition and Analysis

The PCC fusions were manually scanned using the MetaSystems software (MetaSystems, Altlussheim, Germany) X40. γ-H2AX stained PCC were acquired using the automated acquisition module Autocapt software (MetaSystems, version 3.9.1) with a Zeiss Plan-Apochromat 40X and CoolCube 1 Digital High Resolution CCD Camera (Carl Zeiss, Jena, Germany). Telomere and centromere-stained PCC fusions were also acquired with a similar automated acquisition Autocapt module.

For localization of γ-H2AX foci and automatic scoring of chromosome aberrations after TC staining of PCC fusions, the acquired gallery was exported as a series of RGB bitmap images exploitable by any image-processing software. Analysis was performed using the PCC-TCscore and the ISIS software (MetaSystems, version 3.9.1). Using the software tool PCC-TCscore, which is an ImageJ plugin developed in our laboratory, the chromosomes were automatically segmented on the DAPI channel, where CHO chromosomes having two chromatids were excluded. A position map of the telomeres and centromeres was established using the red and green channels. The chromosomes were then defined by classifying each according to its characteristics (number of centromeres and telomeres). Finally, each chromosome showing CA was assigned to one of the following classes: dicentric chromosomes, centric rings, and acentric fragments. However, the results have then to be validated by the operator.

The γ-H2AX foci counting and localization was performed using the ISIS software in parallel to the TC stained images.

### 4.8. Scoring Strategy

All the analyses were performed on three donors with sham irradiation or irradiated at 2 and 4 Gy. For each donor six slides were analysed per irradiation condition and for different time points (3 h/8 h/24 h). The CA were scored and expressed per centromeres for two reasons. First, this calculation excludes bias due to the possible loss of PCC fragments during the cytospin step. Moreover, the number of centromeres does not change with the received dose of IR, while the number of chromosome fragments can increase with dose and acentric fragments evolve until 24 h post-exposure. For the same reason γ-H2AX foci were also reported to the number of centromeres. For the M-FISH analysis, only complete PCC cells (between 45 to 48 chromosome centromeres) were considered.

### 4.9. Statistic Analysis

All the result has been analysed using Statistica software (StatSoft, Inc., Tulsa, OK, USA) for Student test, all samples were considered as independent. For each time point and dose, three donors have been processed in duplicate. For the first time point (3 h), respectively 12789, 6807, and 3951 PCC fragments have been observed for 0, 2, and 4 Gy irradiation. At 8 h post-irradiation, 14793, 10615, and 7449 PCC fragments have been studied respectively for 0, 2, and 4 Gy. For the last time point (24 h), 13893, 9905, and 9984 PCC fragments have been captured for 0, 2, and 4 Gy.

## 5. Conclusions

The novel approach combining the PCC techniques with γ-H2AX foci immunofluorescence staining and chromosome FISH staining allows the deciphering of DNA DSB repair mechanism in parallel with DNA breaks signalling. Here we show that DNA DSB repair occurs in two distinct steps. Within the 3 h post-IR, an early mechanism occurs leading to the majority of chromosome free ends joining, including the induction of CA rearrangement (dicentric chromosomes and chromosomes exchanges). However, about 20% of DNA breaks are correctly repaired in a slower and hardly detectable step where the majority of deleted chromosomes joins acentric fragments to restore chromosome integrity. The multiple staining also reveal that kinetics of DNA repairs and DNA breaks signalling differ since we report an important remaining of γ-H2AX foci several hours after chromosome joining as well as important γ-H2AX signalling in telomere sequences suggesting other functions for γ-H2AX foci.

In regards to biodosimetry, PCC analysis is one of the main cytogenetic techniques to determine individual received dose after unknown irradiation. One of the major advantages of the technique is the absence of cell culture avoiding delay in case of emergency. Here we proved that dicentric chromosomes are formed early after IR and evaluation of doses through dicentric chromosome counting can be done as soon as 3 h post-irradiation. However, the classical approach for PCC consisting in counting total PCC fragments, and we recommend making such a classical analysis at least 24 h post-IR.

## Abbreviations

Abbreviations used in the paper are explained here. Some chromosome aberrations are represented by a scheme. Chromatid is drawing in blue while centromeres are in green and telomeres in red.

**Abbreviations**	**Meaning**	**Scheme**
IR	Irradiation	
CA	Chromosome aberration	
Ac	Acentric fragments	
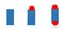
Ac0T	Acentric fragments without telomere	
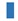
Ac1T	Acentric fragments with 1 telomere	
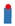
Ac2T	Acentric fragments with 2 telomeres	
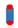
Dic+R	Dicentric and Ring Chromosomes	
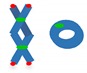
γ-H2AX foci	Phosphorylated form of Histone2AX in foci	
